# Supplementary material for: Molecular Transducers of Physical Activity Consortium (MoTrPAC): Initial Insights into the Dynamic Human Responses to Exercise
Source: bioRxiv. 2026 Mar 5:2026.03.02.705347. Preprint. [Version 1] doi: 10.64898/2026.03.02.705347 (PMC13001330; doi:10.64898/2026.03.02.705347)
Supplement: Supplement 1 — Supplemental Figure 1. Individual handgrip strength and daily step count per day graphed by age. A) Individual maximal handgrip strength at baseline for randomized participants (N=206; 149F, 57M) are graphed by age and compared with normative values from the NIH Toolbox Project (783 females, 449 males)20. Grey area represents 10th-90th percentiles of NIH Toolbox Project database across the adult lifespan. B) Individual average step count per day for females (N=140) and males (N=53) were graphed by age. Data were calculated from average of 5–8 compliant days during baseline Actigraph collection. Supplemental Figure 2. Absolute change in maximal CPET and strength metrics from pre-intervention baseline to post-intervention follow-up. Change in VO2peak (L·min−1) (A), maximal O2 pulse (mL·beat−1) (B), and maximal workload (W) (C) from the cardiopulmonary exercise test (CPET). Change in strength measurements including maximal leg strength [isometric knee extensor strength (Nm)] (D) and maximal hand grip strength (kg) (E). Supplemental Figure 3. Pre-intervention baseline and post-intervention follow-up endurance exercise acute bout parameters. A-C) Exercise intensity shown as (A) percent VO2peak, (B) oxygen uptake (L·min−1), and (C) workload (W). Target exercise intensity range of 65±5% VO2peak is indicated by the dotted lines and grey shading in panel A. D) Average carbohydrate (CHO) and fat utilization during the acute test. Data shown as (kcal·min−1) and calculated as CHO (kcal·min−1) = CHO (g·min−1) * 4.07 (kcal·g−1) and fat (kcal·min−1) = FAT (g·min−1) * 9.75 (kcal·g−1). E) Following the 5 min warm-up, the median (25th-75th percentile) heart rate progression is plotted during the 40 min acute bout over 5 min intervals. Data shown in panels A-D are boxplots and individual observations. Individual observations in panels A-D are 6 min averages of minutes 14–17 with 34–37. Supplemental Figure 4. Venous blood lactate from pre-intervention baseline and post-intervention fol [file media-1.pdf]

Supplemental Figures & Tables

Supplemental Figures
Supplemental Figure 1: Individual handgrip strength and daily step count per day graphed by age. 3
Supplemental Figure 2: Absolute change in maximal CPET and strength metrics from pre-intervention baseline to post-intervention follow-up 4
Supplemental Figure 3: Pre-intervention baseline and post-intervention follow-up endurance exercise acute bout parameters 5
Supplemental Figure 4: Venous blood lactate from pre-intervention baseline and post-intervention follow-up acute endurance and resistance tests 6
Supplemental Figure 5: Pre-intervention baseline and post-intervention follow-up resistance exercise acute bout parameters 7
Supplemental Tables
Supplemental Table 1. Pre-intervention baseline characteristics of participants by sex and age group. 9
Supplemental Table 2. Pre-intervention baseline characteristics of participants who initiated the baseline acute test by randomized intervention group. 12
Supplemental Table 3. Pre-intervention baseline characteristics of participants by baseline acute test initiation status. 15
Supplemental Table 4. Pre-intervention baseline characteristics of participants with post-intervention follow-up data by randomized intervention group. 18
Supplemental Table 5. Pre-intervention baseline endurance exercise acute bout parameters by sex and age group. 21
Supplemental Table 6. Pre-intervention baseline resistance exercise acute bout parameters by age and sex. 23
Supplemental Table 7. Exercise intervention data summary by week. 26
Supplemental Table 8. Baseline and follow-up characteristics of participants with follow-up phenotypic data by randomized intervention group. 28
Supplemental Table 9. Baseline and follow-up endurance exercise acute bout parameters. 31
Supplemental Table 10. Baseline and follow-up resistance exercise acute bout parameters. 32
Supplemental Table 11. Overview of biospecimen collection success for each sample type at baseline and follow-up. 35

Supplemental Figures

Supplemental Figure 1: Individual handgrip strength and daily step count per day graphed by age.

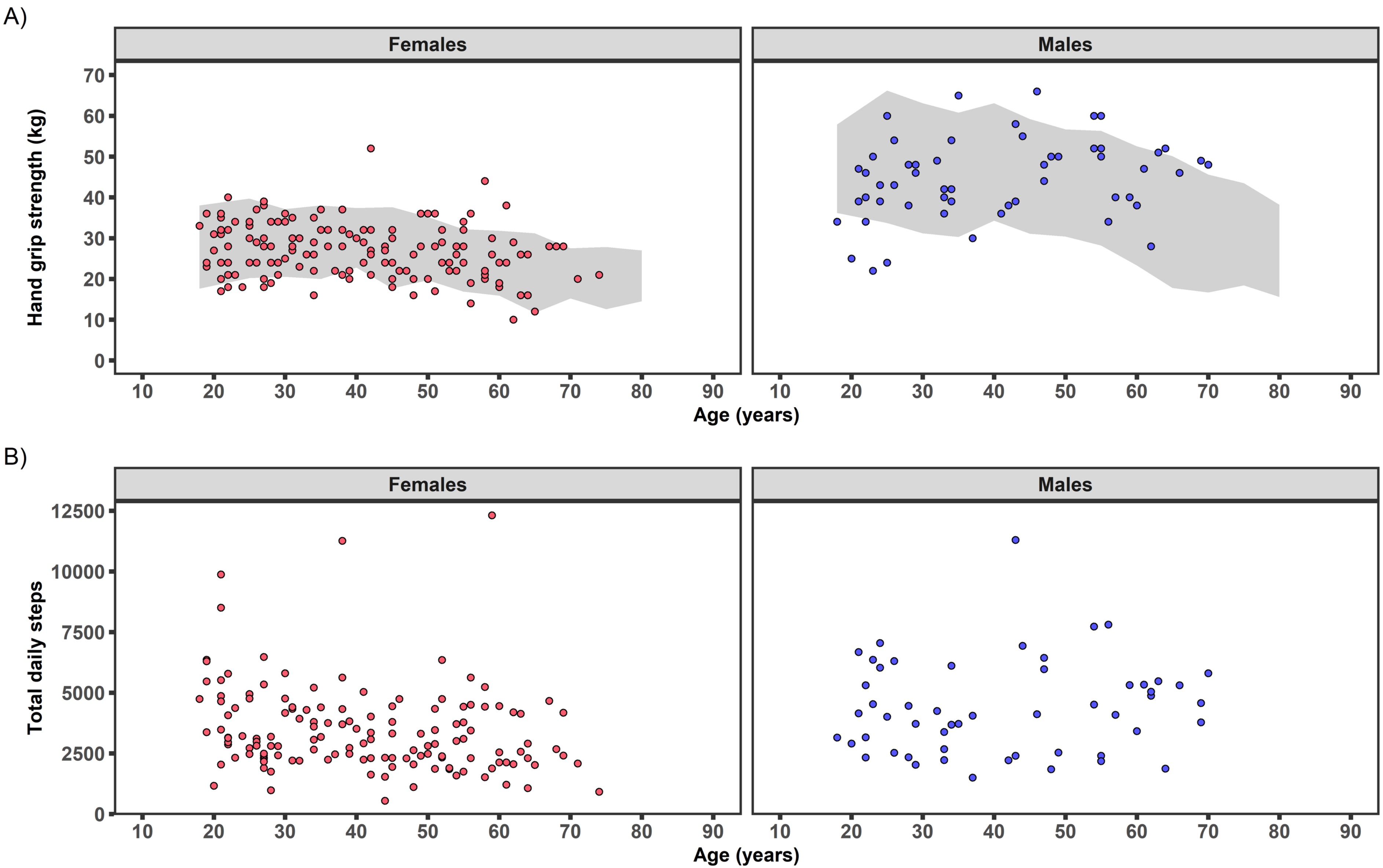

Supplemental Figure 2: Absolute change in maximal CPET and strength metrics from pre-intervention baseline to post-intervention follow-up

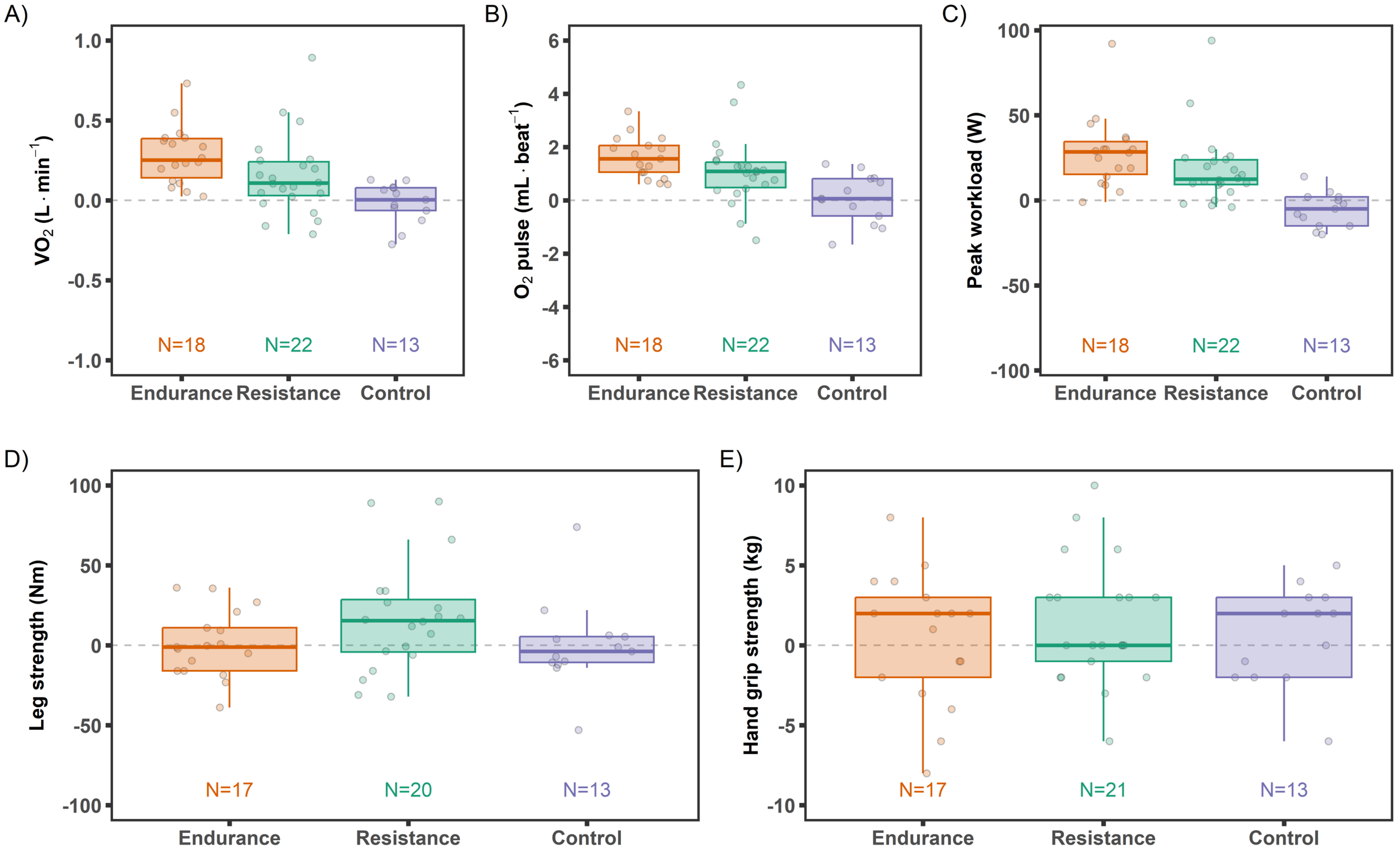

Supplemental Figure 3: Pre-intervention baseline and post-intervention follow-up endurance exercise acute bout parameters

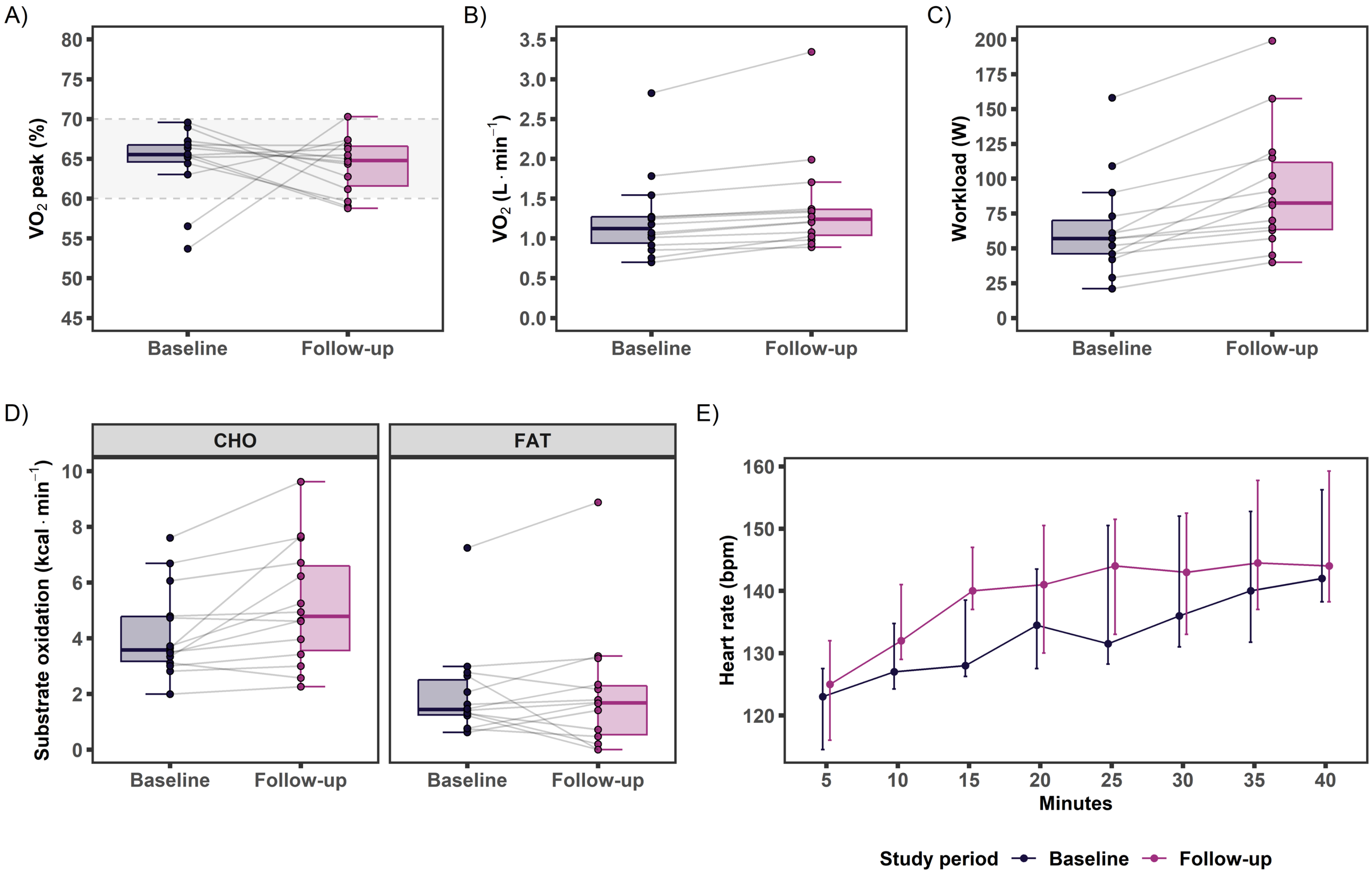

Supplemental Figure 4: Venous blood lactate from pre-intervention baseline and post-intervention follow-up acute endurance and resistance tests

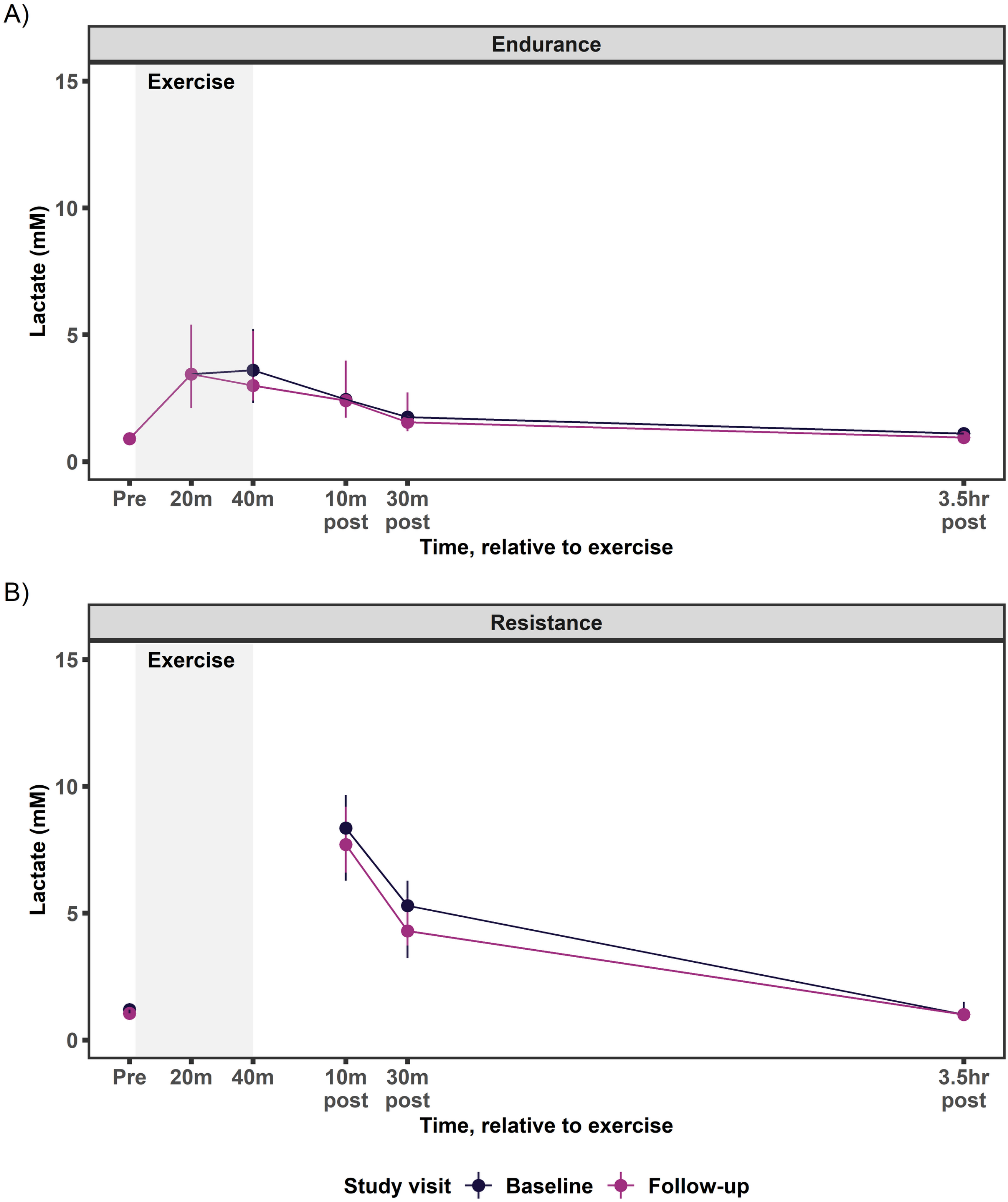

Supplemental Figure 5: Pre-intervention baseline and post-intervention follow-up resistance exercise acute bout parameters

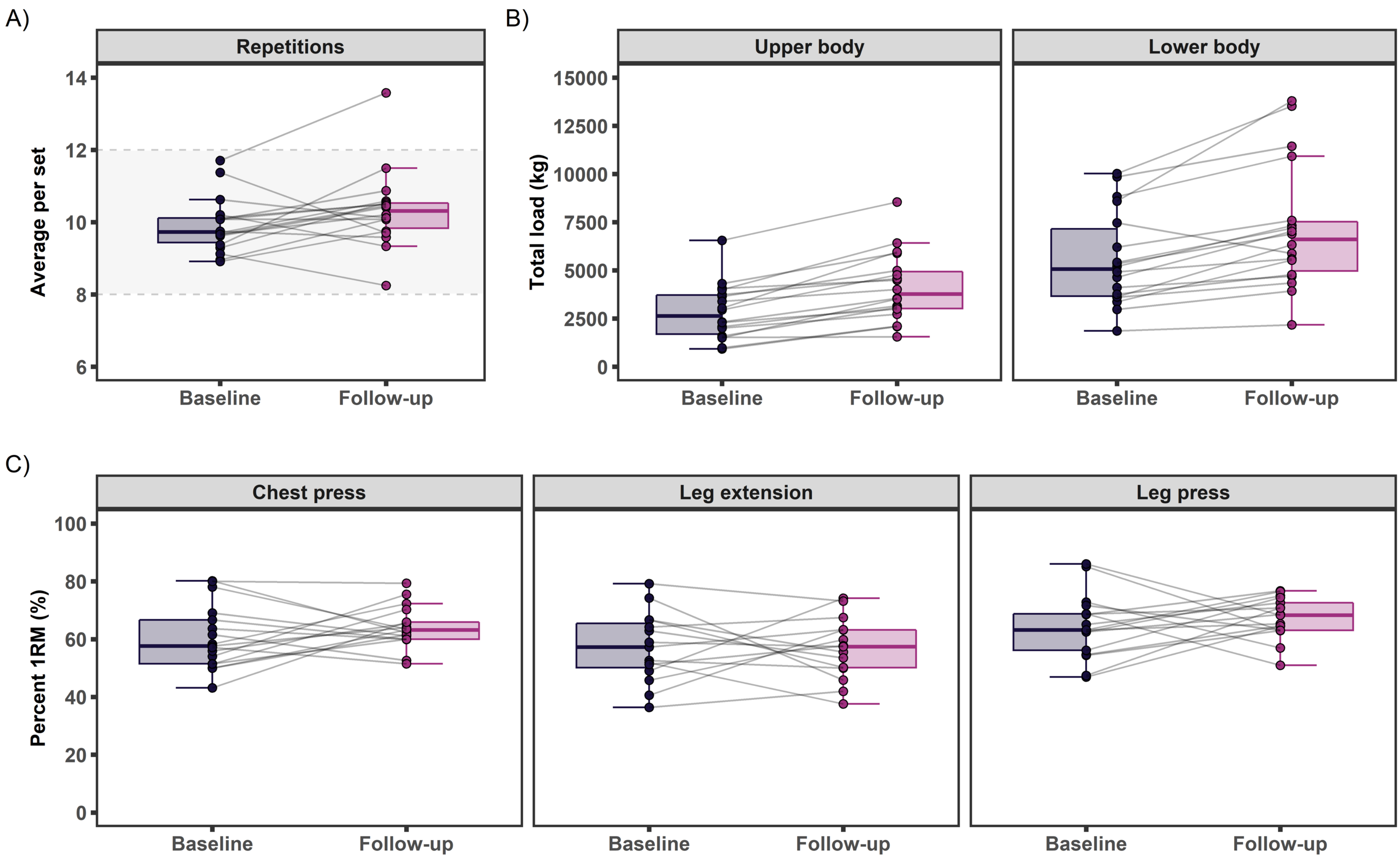

Supplemental Tables

Supplemental Table 1. Pre-intervention baseline characteristics of participants by sex and age group.

Characteristic[1]	Overall N = 206	Females		Males	
		18-39 years N = 75	40+ years N = 74	18-39 years N = 29	40+ years N = 28
Demographics					
Age (years)	39 (27, 54)	27 (22, 33)	53 (45, 60)	26 (23, 33)	55 (47, 62)
Race, %					
African American/Black	17.48	25.33	18.92	3.45	7.14
Asian	4.85	9.33	1.35	6.90	0.00
Caucasian/White	73.30	60.00	77.03	86.21	85.71
Native Hawaiian/Pacific Islander	0.00	0.00	0.00	0.00	0.00
Native American/Alaskan Native	0.49	0.00	1.35	0.00	0.00
Other	1.46	1.33	1.35	3.45	0.00
More than one race	2.43	4.00	0.00	0.00	7.14
Latino, Hispanic or of Spanish origin, %	18.0	21.3	13.5	24.1	14.3
Anthropometrics					
Height (cm)	166.4 (161.2, 173.2)	164.4 (160.6, 168.3)	163.2 (160.0, 167.5)	177.5 (170.5, 182.3)	180.5 (173.8, 183.9)
Weight (kg)	75.1 (64.6, 84.2)	72.3 (61.3, 83.1)	71.1 (62.8, 79.7)	82.1 (71.4, 87.3)	87.2 (81.5, 99.7)
BMI ($kg \cdot m^{-2}$)	27.2 (23.7, 29.8)	27.3 (23.0, 30.7)	26.9 (23.8, 29.6)	25.2 (22.7, 28.2)	27.9 (25.8, 30.9)
Waist circumference (cm)	91 (84, 100)	87 (78, 99)	91 (85, 99)	91 (83, 97)	98 (95, 108)
Resting heart rate (bpm)	62 (56, 67)	63 (57, 70)	64 (58, 68)	60 (55, 64)	59 (51, 63)
Resting systolic BP (mmHg)	116 (107, 124)	109 (104, 116)	119 (111, 126)	118 (116, 121)	127 (113, 136)
Resting diastolic BP (mmHg)	72 (67, 79)	70 (64, 74)	73 (68, 80)	73 (70, 78)	79 (69, 83)
Biomarkers					

Characteristic[1]	Overall N = 206	Females		Males	
		18-39 years N = 75	40+ years N = 74	18-39 years N = 29	40+ years N = 28
HbA1c (%)	5.3 (5.1, 5.5)	5.2 (5.0, 5.4)	5.4 (5.2, 5.6)	5.2 (4.9, 5.3)	5.4 (5.3, 5.5)
Glucose ($mg \cdot dL^{-1}$)	89 (83, 95)	85 (81, 90)	91 (84, 98)	88 (86, 96)	94 (88, 101)
Triglycerides ($mg \cdot dL^{-1}$)	84 (62, 109)	74 (55, 98)	88 (72, 111)	83 (62, 129)	94 (75, 162)
Total cholesterol ($mg \cdot dL^{-1}$)	191 (162, 222)	178 (157, 202)	209 (176, 235)	161 (142, 191)	202 (175, 231)
HDL cholesterol ($mg \cdot dL^{-1}$)	56 (46, 67)	58 (46, 69)	63 (53, 74)	46 (41, 52)	48 (42, 55)
LDL cholesterol ($mg \cdot dL^{-1}$)	115 (91, 140)	104 (87, 133)	124 (103, 149)	100 (83, 113)	136 (107, 152)
VLDL cholesterol ($mg \cdot dL^{-1}$)	17 (12, 24)	16 (11, 21)	19 (13, 25)	18 (12, 27)	19 (16, 27)
Hematocrit (%)	41.5 (39.4, 44.1)	40.5 (38.2, 42.4)	40.7 (38.5, 41.9)	44.7 (43.0, 46.6)	45.1 (43.5, 47.7)
Thyroid stimulating hormone ($mIU \cdot L^{-1}$)	1.7 (1.2, 2.4)	1.6 (1.2, 2.2)	1.7 (1.2, 2.5)	1.7 (1.1, 2.2)	2.0 (1.4, 2.6)
Creatinine ($mg \cdot dL^{-1}$)	0.80 (0.68, 0.90)	0.72 (0.64, 0.80)	0.73 (0.67, 0.85)	0.97 (0.81, 1.08)	0.98 (0.90, 1.13)
eGFR ($mL \cdot min^{-1} \cdot 1.73m^{-2}$)	103 (90, 115)	114 (101, 124)	95 (80, 105)	110 (97, 125)	92 (77, 100)
Cardiopulmonary Exercise Testing					
VO_2peak ($L \cdot min^{-1}$)	1.68 (1.42, 2.17)	1.70 (1.52, 1.97)	1.40 (1.21, 1.55)	2.80 (2.24, 2.93)	2.54 (2.12, 2.86)
VO_2peak ($mL \cdot kg^{-1} \cdot min^{-1}$)	24.0 (19.2, 28.7)	24.4 (20.5, 28.4)	19.0 (17.1, 22.3)	34.6 (30.5, 39.1)	26.9 (25.3, 32.4)
Peak ventilation ($L \cdot min^{-1}$)	53.9 (44.2, 70.8)	53.6 (45.2, 61.2)	45.0 (38.7, 53.6)	81.1 (65.3, 94.2)	82.4 (65.6, 99.5)
Peak O_2 pulse ($mL \cdot beat^{-1}$)	9.6 (8.5, 12.1)	9.5 (8.6, 10.9)	8.7 (7.5, 9.4)	14.0 (11.7, 16.1)	14.4 (12.5, 17.3)
Peak RER	1.17 (1.12, 1.22)	1.16 (1.12, 1.20)	1.18 (1.10, 1.24)	1.16 (1.12, 1.21)	1.19 (1.15, 1.24)
Peak workload (W)	143 (115, 179)	145 (125, 164)	112 (99, 127)	209 (178, 229)	197 (180, 234)

Characteristic[1]	Overall N = 206	Females		Males	
		18-39 years N = 75	40+ years N = 74	18-39 years N = 29	40+ years N = 28
Peak heart rate (bpm)	176 (163, 187)	182 (174, 190)	166 (157, 173)	193 (185, 200)	168 (161, 179)
Systolic BP at termination (mmHg)	162 (150, 182)	158 (145, 170)	163 (152, 184)	160 (152, 180)	187 (170, 204)
Diastolic BP at termination (mmHg)	78 (70, 84)	74 (64, 80)	80 (72, 86)	78 (66, 88)	81 (73, 88)
Overall RPE at termination	19 (17, 19)	19 (17, 19)	19 (17, 19)	19 (18, 20)	18 (17, 19)
Physical measures					
Leg strength (Nm)[2]	143 (112, 193)	141 (115, 169)	114 (97, 138)	196 (164, 239)	202 (171, 231)
Hand grip strength (kg)[3]	30 (24, 38)	28 (24, 33)	26 (21, 29)	42 (36, 48)	49 (40, 52)

[1]Table values are median (25th, 75th percentile) or percentage for continuous and categorical variables, respectively

[2]The maximum of three peak torques obtained through three maximal voluntary contraction trials lasting 5 seconds each

[3]The maximum of three grip strength trials which occur after one submaximal practice trial to familiarize the participant with the feel of the instrument

BMI, body mass index; BP, blood pressure; HDL, high-density lipoprotein; HbA1c, hemoglobin A1c; LDL, low-density lipoprotein; RPE, rating of perceived exertion; VLDL, very low-density lipoprotein; VO_2, volume of oxygen; W, watts; eGFR, estimated glomerular filtration rate

Supplemental Table 2. Pre-intervention baseline characteristics of participants who initiated the baseline acute test by randomized intervention group.

Characteristic[1]	Overall N = 176	Intervention Group		
		Endurance N = 66	Resistance N = 73	Control N = 37
Demographics				
Age (years)	41 (27, 54)	42 (28, 53)	38 (27, 54)	45 (31, 54)
Sex, %				
Females	72.2	71.2	67.1	83.8
Males	27.8	28.8	32.9	16.2
Race, %				
African American/Black	18.18	22.73	15.07	16.22
Asian	5.11	7.58	5.48	0.00
Caucasian/White	72.73	65.15	75.34	81.08
Native Hawaiian/Pacific Islander	0.00	0.00	0.00	0.00
Native American/Alaskan Native	0.57	0.00	1.37	0.00
Other	1.70	3.03	1.37	0.00
More than one race	1.70	1.52	1.37	2.70
Latino, Hispanic or of Spanish origin, %	18.2	19.7	19.2	13.5
Anthropometrics				
Height (cm)	166.7 (161.2, 173.6)	166.1 (162.4, 174.5)	166.7 (161.2, 173.4)	166.9 (160.9, 171.4)
Weight (kg)	76.0 (65.0, 85.0)	76.9 (64.6, 86.5)	77.1 (65.8, 85.1)	73.5 (62.5, 82.5)
BMI ($kg \cdot m^{-2}$)	27.3 (23.8, 29.9)	26.8 (23.9, 30.7)	27.6 (23.8, 30.1)	26.1 (23.7, 28.4)
Waist circumference (cm)	92 (84, 100)	93 (85, 99)	92 (84, 101)	90 (79, 96)
Resting heart rate (bpm)	62 (56, 67)	62 (55, 68)	61 (57, 67)	64 (58, 66)
Resting systolic BP (mmHg)	116 (107, 124)	116 (108, 125)	117 (106, 124)	113 (106, 121)
Resting diastolic BP (mmHg)	72 (67, 78)	73 (68, 79)	72 (65, 78)	72 (68, 78)

Characteristic[1]	Overall N = 176	Intervention Group		
		Endurance N = 66	Resistance N = 73	Control N = 37
Biomarkers				
HbA1c (%)	5.3 (5.1, 5.5)	5.3 (5.1, 5.5)	5.3 (5.1, 5.5)	5.3 (5.2, 5.4)
Glucose (mg·dL^{-1})	89 (84, 95)	89 (81, 92)	88 (84, 94)	90 (87, 96)
Triglycerides (mg·dL^{-1})	84 (64, 110)	84 (62, 117)	88 (72, 127)	78 (55, 89)
Total cholesterol (mg·dL^{-1})	191 (162, 224)	186 (161, 225)	194 (165, 218)	188 (162, 234)
HDL cholesterol (mg·dL^{-1})	56 (46, 67)	56 (45, 68)	54 (45, 63)	61 (52, 68)
LDL cholesterol (mg·dL^{-1})	116 (91, 140)	116 (91, 143)	116 (99, 137)	115 (91, 158)
VLDL cholesterol (mg·dL^{-1})	17 (13, 24)	17 (12, 26)	18 (15, 26)	16 (11, 21)
Hematocrit (%)	41.5 (39.1, 43.9)	41.9 (39.7, 44.0)	41.6 (38.3, 44.6)	41.0 (39.0, 42.7)
Thyroid stimulating hormone (mIU·L^{-1})	1.7 (1.3, 2.4)	1.8 (1.4, 2.6)	1.5 (1.1, 2.2)	1.9 (1.3, 2.5)
Creatinine (mg·dL^{-1})	0.80 (0.69, 0.90)	0.80 (0.70, 0.90)	0.80 (0.70, 0.97)	0.80 (0.68, 0.86)
eGFR (mL·min^{-1}·1.73m^{-2})	103 (90, 115)	104 (92, 116)	102 (88, 114)	103 (89, 112)
Cardiopulmonary Exercise Testing				
VO_2peak (L·min^{-1})	1.69 (1.41, 2.16)	1.74 (1.42, 2.20)	1.71 (1.42, 2.30)	1.58 (1.37, 2.03)
VO_2peak (mL·kg^{-1}·min^{-1})	23.6 (18.9, 28.7)	23.0 (19.8, 28.9)	24.1 (18.9, 28.4)	22.8 (17.2, 28.1)
Peak ventilation (L·min^{-1})	53.9 (44.1, 70.9)	55.4 (47.7, 68.3)	53.8 (43.8, 75.5)	50.4 (42.0, 62.0)
Peak O_2 pulse (mL·beat^{-1})	9.7 (8.4, 12.2)	9.9 (8.6, 12.2)	9.8 (8.2, 12.5)	9.4 (8.4, 10.9)
Peak RER	1.17 (1.12, 1.22)	1.18 (1.14, 1.22)	1.16 (1.09, 1.21)	1.16 (1.12, 1.24)
Peak workload (W)	143 (115, 180)	142 (115, 185)	150 (115, 185)	143 (115, 170)

Characteristic[1]	Overall<br>N = 176	Intervention Group		
		Endurance<br>N = 66	Resistance<br>N = 73	Control<br>N = 37
Peak heart rate (bpm)	175<br>(162, 187)	175<br>(160, 187)	175<br>(166, 185)	174<br>(161, 185)
Systolic BP at termination (mmHg)	162<br>(150, 184)	165<br>(152, 188)	160<br>(150, 180)	160<br>(150, 182)
Diastolic BP at termination (mmHg)	78<br>(70, 86)	79<br>(70, 88)	78<br>(66, 84)	80<br>(72, 84)
Overall RPE at termination	19<br>(17, 19)	19<br>(17, 19)	19<br>(17, 19)	19<br>(18, 19)
Physical measures				
Leg strength (Nm)[2]	141<br>(108, 193)	135<br>(103, 201)	149<br>(116, 196)	134<br>(106, 156)
Hand grip strength (kg)[3]	30<br>(24, 38)	29<br>(24, 37)	30<br>(24, 40)	29<br>(24, 34)

[1]*Table values are median (25th, 75th percentile) or percentage for continuous and categorical variables, respectively*

[2]*The maximum of three peak torques obtained through three maximal voluntary contraction trials lasting 5 seconds each*

[3]*The maximum of three grip strength trials which occur after one submaximal practice trial to familiarize the participant with the feel of the instrument*

BMI, body mass index; BP, blood pressure; HDL, high-density lipoprotein; HbA1c, hemoglobin A1c; LDL, low-density lipoprotein; RPE, rating of perceived exertion; VLDL, very low-density lipoprotein; VO_2*, volume of oxygen; W, watts; eGFR, estimated glomerular filtration rate*

Supplemental Table 3. Pre-intervention baseline characteristics of participants by baseline acute test initiation status.

Characteristic[1]	Overall<br>N = 206	Baseline acute test initiated	
		No<br>N = 30	Yes<br>N = 176
Demographics			
Age (years)	39<br>(27, 54)	32<br>(26, 55)	41<br>(27, 54)
Sex, %			
Females	72.3	73.3	72.2
Males	27.7	26.7	27.8
Race, %			
African American/Black	17.48	13.33	18.18
Asian	4.85	3.33	5.11
Caucasian/White	73.30	76.67	72.73
Native Hawaiian/Pacific Islander	0.00	0.00	0.00
Native American/Alaskan Native	0.49	0.00	0.57
Other	1.46	0.00	1.70
More than one race	2.43	6.67	1.70
Latino, Hispanic or of Spanish origin, %	18.0	16.7	18.2
Anthropometrics			
Height (cm)	166.4<br>(161.2, 173.2)	164.4<br>(161.3, 171.4)	166.7<br>(161.2, 173.6)
Weight (kg)	75.1<br>(64.6, 84.2)	73.3<br>(63.7, 83.2)	76.0<br>(65.0, 85.0)
BMI ($kg \cdot m^{-2}$)	27.2<br>(23.7, 29.8)	25.6<br>(23.6, 28.8)	27.3<br>(23.8, 29.9)
Waist circumference (cm)	91<br>(84, 100)	87<br>(81, 97)	92<br>(84, 100)
Resting heart rate (bpm)	62<br>(56, 67)	63<br>(59, 66)	62<br>(56, 67)
Resting systolic BP (mmHg)	116<br>(107, 124)	118<br>(111, 123)	116<br>(107, 124)
Resting diastolic BP (mmHg)	72<br>(67, 79)	72<br>(68, 80)	72<br>(67, 78)

Characteristic[1]	Overall<br>N = 206	Baseline acute test initiated	
		No<br>N = 30	Yes<br>N = 176
Biomarkers			
HbA1c (%)	5.3<br>(5.1, 5.5)	5.2<br>(5.0, 5.4)	5.3<br>(5.1, 5.5)
Glucose (mg·dL^{-1})	89<br>(83, 95)	88<br>(82, 99)	89<br>(84, 95)
Triglycerides (mg·dL^{-1})	84<br>(62, 109)	86<br>(54, 106)	84<br>(64, 110)
Total cholesterol (mg·dL^{-1})	191<br>(162, 222)	190<br>(165, 212)	191<br>(162, 224)
HDL cholesterol (mg·dL^{-1})	56<br>(46, 67)	59<br>(45, 68)	56<br>(46, 67)
LDL cholesterol (mg·dL^{-1})	115<br>(91, 140)	106<br>(87, 139)	116<br>(91, 140)
VLDL cholesterol (mg·dL^{-1})	17<br>(12, 24)	19<br>(11, 23)	17<br>(13, 24)
Hematocrit (%)	41.5<br>(39.4, 44.1)	41.9<br>(40.5, 44.9)	41.5<br>(39.1, 43.9)
Thyroid stimulating hormone (mIU·L^{-1})	1.7<br>(1.2, 2.4)	1.3<br>(1.0, 2.4)	1.7<br>(1.3, 2.4)
Creatinine (mg·dL^{-1})	0.80<br>(0.68, 0.90)	0.80<br>(0.68, 0.95)	0.80<br>(0.69, 0.90)
eGFR (mL·min^{-1}·1.73m^{-2})	103<br>(90, 115)	101<br>(90, 117)	103<br>(90, 115)
Cardiopulmonary Exercise Testing			
VO_2peak (L·min^{-1})	1.68<br>(1.42, 2.17)	1.68<br>(1.51, 2.19)	1.69<br>(1.41, 2.16)
VO_2peak (mL·kg^{-1}·min^{-1})	24.0<br>(19.2, 28.7)	26.1<br>(20.5, 30.6)	23.6<br>(18.9, 28.7)
Peak ventilation (L·min^{-1})	53.9<br>(44.2, 70.8)	54.4<br>(44.8, 66.6)	53.9<br>(44.1, 70.9)
Peak O_2 pulse (mL·beat^{-1})	9.6<br>(8.5, 12.1)	9.5<br>(8.9, 11.9)	9.7<br>(8.4, 12.2)
Peak RER	1.17<br>(1.12, 1.22)	1.16<br>(1.13, 1.24)	1.17<br>(1.12, 1.22)
Peak workload (W)	143<br>(115, 179)	140<br>(128, 177)	143<br>(115, 180)

**Characteristic**[1]	**Overall**<br>**N = 206**	**Baseline acute test initiated**	
		**No**<br>**N = 30**	**Yes**<br>**N = 176**
Peak heart rate (bpm)	176<br>(163, 187)	182<br>(174, 187)	175<br>(162, 187)
Systolic BP at termination (mmHg)	162<br>(150, 182)	163<br>(150, 182)	162<br>(150, 184)
Diastolic BP at termination (mmHg)	78<br>(70, 84)	73<br>(66, 82)	78<br>(70, 86)
Overall RPE at termination	19<br>(17, 19)	19<br>(17, 20)	19<br>(17, 19)
Physical measures			
Leg strength (Nm)[2]	143<br>(112, 193)	146<br>(125, 231)	141<br>(108, 193)
Hand grip strength (kg)[3]	30<br>(24, 38)	29<br>(22, 38)	30<br>(24, 38)

[1]Table values are median (25th, 75th percentile) or percentage for continuous and categorical variables, respectively

[2]The maximum of three peak torques obtained through three maximal voluntary contraction trials lasting 5 seconds each

[3]The maximum of three grip strength trials which occur after one submaximal practice trial to familiarize the participant with the feel of the instrument

BMI, body mass index; BP, blood pressure; HDL, high-density lipoprotein; HbA1c, hemoglobin A1c; LDL, low-density lipoprotein; RPE, rating of perceived exertion; VLDL, very low-density lipoprotein; VO_2, volume of oxygen; W, watts; eGFR, estimated glomerular filtration rate

Supplemental Table 4. Pre-intervention baseline characteristics of participants with post-intervention follow-up data by randomized intervention group.

Characteristic[1]	Overall N = 44	Intervention Group		
		Endurance N = 14	Resistance N = 18	Control N = 12
Demographics				
Age (years)	42 (27, 58)	32 (25, 59)	36 (26, 55)	54 (48, 61)
Sex, %				
Females	75.0	78.6	72.2	75.0
Males	25.0	21.4	27.8	25.0
Race, %				
African American/Black	13.64	28.57	5.56	8.33
Asian	6.82	7.14	11.11	0.00
Caucasian/White	75.00	57.14	77.78	91.67
Native Hawaiian/Pacific Islander	0.00	0.00	0.00	0.00
Native American/Alaskan Native	2.27	0.00	5.56	0.00
Other	2.27	7.14	0.00	0.00
More than one race	0.00	0.00	0.00	0.00
Latino, Hispanic or of Spanish origin, %	27.3	28.6	27.8	25.0
Anthropometrics				
Height (cm)	165.7 (159.5, 171.3)	167.1 (157.3, 173.8)	164.7 (158.6, 172.7)	164.9 (160.0, 169.1)
Weight (kg)	71.2 (63.8, 81.7)	71.0 (63.0, 89.4)	72.1 (65.8, 81.3)	70.8 (64.9, 78.1)
BMI ($kg \cdot m^{-2}$)	26.7 (24.2, 29.1)	26.1 (23.7, 29.6)	27.4 (24.3, 29.8)	26.4 (24.5, 28.1)
Waist circumference (cm)	90 (83, 99)	91 (82, 98)	90 (87, 101)	89 (81, 96)
Resting heart rate (bpm)	60 (52, 65)	59 (52, 67)	60 (55, 63)	60 (51, 67)
Resting systolic BP (mmHg)	116 (109, 123)	117 (116, 124)	111 (107, 119)	116 (109, 131)
Resting diastolic BP (mmHg)	72 (65, 78)	74 (69, 79)	70 (62, 75)	71 (68, 81)

Characteristic[1]	Overall N = 44	Intervention Group		
		Endurance N = 14	Resistance N = 18	Control N = 12
Biomarkers				
HbA1c (%)	5.3 (5.2, 5.5)	5.3 (5.1, 5.6)	5.3 (5.1, 5.5)	5.3 (5.2, 5.5)
Glucose (mg·dL^{-1})	90 (84, 95)	89 (79, 97)	91 (84, 94)	92 (87, 97)
Triglycerides (mg·dL^{-1})	84 (68, 109)	88 (69, 109)	93 (79, 131)	70 (52, 88)
Total cholesterol (mg·dL^{-1})	190 (167, 225)	186 (174, 225)	194 (167, 218)	188 (164, 246)
HDL cholesterol (mg·dL^{-1})	65 (50, 74)	69 (49, 72)	60 (43, 74)	67 (59, 78)
LDL cholesterol (mg·dL^{-1})	114 (90, 135)	118 (91, 135)	112 (90, 128)	111 (79, 161)
VLDL cholesterol (mg·dL^{-1})	15 (11, 25)	15 (11, 26)	17 (14, 20)	14 (11, 21)
Hematocrit (%)	41.65 (39.50, 42.70)	41.30 (39.50, 43.00)	41.80 (39.90, 42.60)	41.15 (39.50, 43.45)
Thyroid stimulating hormone (mIU·L^{-1})	1.7 (1.2, 2.4)	1.8 (1.6, 2.4)	1.5 (1.1, 2.2)	1.7 (0.8, 2.5)
Creatinine (mg·dL^{-1})	0.80 (0.69, 0.90)	0.75 (0.60, 0.90)	0.81 (0.70, 0.90)	0.80 (0.67, 0.91)
eGFR (mL·min^{-1}·1.73m^{-2})	103 (93, 113)	114 (96, 123)	102 (96, 110)	102 (81, 105)
Cardiopulmonary Exercise Testing				
VO_2peak (L·min^{-1})	1.60 (1.29, 2.18)	1.74 (1.42, 1.95)	1.59 (1.26, 2.35)	1.52 (1.22, 1.89)
VO_2peak (mL·kg^{-1}·min^{-1})	22.6 (18.5, 28.8)	22.6 (21.4, 28.7)	23.8 (17.4, 31.6)	21.9 (17.9, 27.6)
Peak ventilation (L·min^{-1})	51.5 (42.6, 66.4)	53.4 (48.8, 58.6)	51.5 (37.9, 77.7)	50.2 (40.8, 68.6)
Peak O_2 pulse (mL·beat^{-1})	9.3 (8.0, 11.8)	9.9 (8.6, 11.5)	9.1 (7.5, 12.9)	8.8 (7.9, 11.5)
Peak RER	1.16 (1.10, 1.21)	1.18 (1.16, 1.21)	1.12 (1.07, 1.20)	1.20 (1.13, 1.27)
Peak workload (W)	128 (105, 179)	147 (120, 184)	124 (105, 185)	128 (105, 152)

Characteristic[1]	Overall N = 44	Intervention Group		
		Endurance N = 14	Resistance N = 18	Control N = 12
Peak heart rate (bpm)	175 (161, 188)	182 (163, 196)	177 (162, 187)	168 (158, 180)
Systolic BP at termination (mmHg)	162 (150, 192)	166 (158, 192)	154 (150, 194)	182 (143, 189)
Diastolic BP at termination (mmHg)	81 (73, 91)	80 (76, 94)	78 (64, 90)	85 (81, 90)
Overall RPE at termination	19 (17, 19)	19 (17, 19)	17 (17, 19)	19 (17, 19)
Physical measures				
Leg strength (Nm)[2]	138 (125, 175)	137 (121, 167)	139 (111, 192)	141 (131, 170)
Hand grip strength (kg)[3]	26 (22, 34)	24 (22, 29)	26 (20, 33)	27 (23, 36)

[1]*Table values are median (25th, 75th percentile) or percentage for continuous and categorical variables, respectively*

[2]*The maximum of three peak torques obtained through three maximal voluntary contraction trials lasting 5 seconds each*

[3]*The maximum of three grip strength trials which occur after one submaximal practice trial to familiarize the participant with the feel of the instrument*

BMI, body mass index; BP, blood pressure; HDL, high-density lipoprotein; HbA1c, hemoglobin A1c; LDL, low-density lipoprotein; RPE, rating of perceived exertion; VLDL, very low-density lipoprotein; VO_2, volume of oxygen; W, watts; eGFR, estimated glomerular filtration rate

Supplemental Table 5. Pre-intervention baseline endurance exercise acute bout parameters by sex and age group.

Variable	Overall N = 64	Females		Males	
		18-39 years N = 20	40+ years N = 26	18-39 years N = 8	40+ years N = 10
Median (25th, 75th percentile)					
Workload (W)	61 (47, 88)	62 (57, 75)	47 (39, 55)	94 (89, 106)	105 (94, 108)
Total Work (kJ)	141 (110, 210)	146 (127, 178)	113 (93, 133)	226 (214, 248)	244 (214, 260)
RPM	71 (66, 75)	73 (69, 77)	70 (65, 72)	72 (71, 74)	72 (68, 78)
Age and sex adjusted mean (95% confidence interval)					
VO_2 (% peak)	64.8 (63.7, 65.9)	64.9 (62.9, 66.8)	63.9 (62.2, 65.6)	65.9 (62.9, 69.0)	65.8 (63.1, 68.5)
VO_2 ($L \cdot min^{-1}$)	1.17 (1.08, 1.28)	1.16 (1.05, 1.29)	0.89 (0.81, 0.96)	1.75 (1.54, 2.08)	1.67 (1.41, 1.96)
VO_2 ($mL \cdot kg^{-1} \cdot min^{-1}$)	15.5 (14.5, 16.8)	15.5 (14.3, 17.1)	12.4 (11.4, 13.5)	23.7 (21.5, 27.0)	18.9 (16.4, 21.9)
Carbohydrate ($g \cdot min^{-1}$)	1.08 (1.01, 1.16)	1.00 (0.88, 1.13)	0.79 (0.68, 0.90)	1.63 (1.43, 1.83)	1.58 (1.40, 1.76)
Fat ($g \cdot min^{-1}$)	0.2 (0.1, 0.2)	0.2 (0.1, 0.2)	0.1 (0.1, 0.2)	0.2 (0.1, 0.4)	0.2 (0.1, 0.3)
Carbohydrate ($kcals \cdot min^{-1}$)	4.1 (3.7, 4.5)	3.9 (3.3, 4.5)	3.1 (2.8, 3.4)	6.4 (5.2, 7.6)	6.3 (5.6, 7.1)
Fat ($kcals \cdot min^{-1}$)	1.5 (1.2, 1.8)	1.6 (1.1, 2.2)	1.2 (0.8, 1.5)	1.8 (0.8, 3.5)	1.7 (0.8, 3.0)
Carbohydrate (%)	72 (68, 76)	69 (62, 77)	71 (65, 78)	76 (64, 88)	77 (66, 88)
VE ($L \cdot min^{-1}$)	29.4 (27.6, 31.4)	28.6 (26.4, 31.0)	24.0 (22.5, 25.3)	39.4 (33.8, 46.4)	41.4 (34.9, 47.8)
RER	0.92 (0.90, 0.93)	0.91 (0.88, 0.93)	0.92 (0.90, 0.94)	0.93 (0.89, 0.96)	0.93 (0.90, 0.96)
Heart rate (bpm)	141 (137, 144)	148 (141, 154)	131 (125, 136)	160 (150, 169)	131 (122, 140)
Heart rate reserve (%)	70 (67, 72)	71 (66, 76)	68 (64, 72)	72 (65, 80)	68 (61, 74)
O_2 Pulse ($mL \cdot beat^{-1}$)	8.4 (7.8, 9.1)	7.9 (7.2, 8.7)	6.9 (6.2, 7.5)	11.0 (9.4, 13.2)	12.9 (10.8, 15.0)

Variable	Overall N = 64	Females		Males	
		18-39 years N = 20	40+ years N = 26	18-39 years N = 8	40+ years N = 10
RER, respiratory exchange ratio; RPM, revolutions per minute; VE, ventilation; VO2, volume of oxygen; W, watts					

Supplemental Table 6. Pre-intervention baseline resistance exercise acute bout parameters by age and sex.

Characteristic[1]	Overall N = 73	Females		Males	
		18-39 years N = 26	40+ years N = 23	18-39 years N = 13	40+ years N = 11
Acute bout duration (minutes)	56 (51, 59)	56 (51, 59)	55 (52, 59)	51 (50, 60)	58 (56, 64)
1RM					
Leg press (kg)	114 (85, 143)	109 (86, 132)	82 (68, 107)	163 (127, 259)	147 (125, 164)
Chest press (kg)	36 (27, 56)	31 (27, 45)	25 (20, 34)	77 (50, 79)	64 (49, 73)
Leg extension (kg)	70 (50, 91)	73 (59, 84)	48 (36, 64)	96 (76, 119)	87 (64, 107)
1RM, normalized					
Leg press ($kg \cdot bw^{-1}$)	1.47 (1.18, 1.78)	1.52 (1.17, 1.77)	1.24 (0.98, 1.32)	1.98 (1.64, 3.47)	1.64 (1.37, 1.68)
Chest press ($kg \cdot bw^{-1}$)	0.50 (0.36, 0.69)	0.45 (0.36, 0.58)	0.35 (0.27, 0.43)	0.83 (0.64, 1.08)	0.67 (0.58, 0.83)
Leg extension ($kg \cdot bw^{-1}$)	0.93 (0.67, 1.12)	0.93 (0.80, 1.09)	0.63 (0.53, 0.87)	1.19 (1.01, 1.54)	0.87 (0.73, 1.27)
Percent 1RM					
Leg press ($kg \cdot 1RM^{-1}$)	72 (64, 76)	72 (64, 76)	73 (68, 77)	68 (63, 73)	66 (64, 74)
Chest press ($kg \cdot 1RM^{-1}$)	62 (54, 71)	62 (56, 67)	64 (54, 78)	64 (56, 78)	59 (54, 71)
Leg extension ($kg \cdot 1RM^{-1}$)	59 (51, 64)	56 (50, 63)	60 (52, 65)	57 (49, 63)	63 (57, 67)
Average resistance					
Chest press (kg)	22 (17, 36)	18 (16, 29)	17 (9, 20)	41 (32, 60)	34 (29, 55)
Overhead press (kg)	9 (6, 15)	9 (6, 12)	6 (5, 12)	19 (11, 26)	14 (6, 20)
Seated row (kg)	26 (20, 34)	22 (18, 27)	22 (17, 27)	39 (27, 43)	42 (38, 50)
Triceps extension (kg)	20 (15, 29)	17 (9, 20)	20 (12, 29)	27 (18, 40)	34 (26, 42)

Characteristic[1]	Overall N = 73	Females		Males	
		18-39 years N = 26	40+ years N = 23	18-39 years N = 13	40+ years N = 11
Biceps curl (kg)	12 (8, 23)	9 (5, 14)	11 (7, 16)	20 (11, 33)	28 (23, 32)
Leg press (kg)	81 (60, 101)	73 (61, 93)	59 (51, 82)	102 (91, 145)	99 (88, 118)
Leg curl (kg)	39 (29, 51)	39 (27, 45)	33 (22, 37)	52 (45, 68)	49 (43, 61)
Leg extension (kg)	40 (27, 54)	36 (27, 47)	26 (21, 39)	54 (45, 66)	57 (47, 69)
Average repetitions per set					
Chest press	9.00 (8.33, 10.00)	8.83 (8.33, 10.00)	9.33 (8.67, 10.00)	9.33 (7.67, 9.67)	8.67 (8.00, 9.67)
Overhead press	9.33 (8.33, 10.00)	9.17 (8.33, 10.00)	9.67 (8.33, 10.33)	8.67 (8.00, 9.33)	9.67 (8.67, 10.00)
Seated row	10.00 (9.00, 10.33)	9.83 (8.67, 10.00)	10.33 (10.00, 11.00)	9.33 (9.00, 10.00)	9.67 (8.67, 10.00)
Triceps extension	10.00 (9.33, 11.00)	10.00 (9.33, 11.33)	10.00 (9.67, 11.00)	9.67 (9.33, 10.33)	10.00 (9.00, 10.67)
Biceps curl	9.67 (8.67, 10.00)	9.67 (8.67, 10.33)	9.67 (9.00, 10.00)	9.33 (7.67, 10.33)	8.67 (8.33, 10.00)
Leg press	10.33 (9.67, 11.67)	10.67 (10.00, 12.00)	10.00 (9.33, 11.00)	10.00 (9.00, 11.00)	11.17 (10.00, 12.00)
Leg curl	10.00 (8.83, 10.50)	9.33 (8.33, 10.00)	10.00 (9.00, 10.67)	9.33 (9.00, 10.33)	10.00 (9.67, 10.67)
Leg extension	9.67 (8.67, 10.33)	9.83 (9.00, 10.67)	9.67 (8.33, 10.00)	9.33 (8.67, 10.00)	9.00 (8.33, 10.00)
Overall	9.7 (9.3, 10.1)	9.8 (9.3, 10.3)	9.8 (9.3, 10.1)	9.4 (8.9, 10.0)	9.7 (9.0, 9.8)
Total load					
Upper body (kg)	2,756 (1,871, 3,785)	2,072 (1,731, 2,756)	2,353 (1,645, 2,987)	4,064 (3,040, 4,797)	4,175 (3,710, 5,241)
Lower body (kg)	4,892 (3,681, 6,195)	4,686 (3,520, 5,825)	3,758 (3,234, 4,567)	7,466 (5,166, 8,836)	6,167 (5,674, 7,119)
Combined (kg)	7,537 (5,783, 9,840)	6,632 (5,368, 8,292)	5,984 (5,211, 6,895)	11,530 (8,480, 13,856)	10,637 (9,362, 12,408)
Total load, normalized					

Characteristic[1]	Overall N = 73	Females		Males	
		18-39 years N = 26	40+ years N = 23	18-39 years N = 13	40+ years N = 11
Upper body (kg·bw^{-1})	38 (27, 45)	29 (22, 41)	34 (25, 40)	46 (42, 62)	49 (45, 53)
Lower body (kg·bw^{-1})	62 (50, 77)	63 (55, 77)	50 (43, 62)	85 (67, 111)	69 (62, 81)
Combined (kg·bw^{-1})	101 (83, 116)	94 (82, 115)	83 (73, 100)	131 (110, 156)	113 (110, 132)

[1]Table values are median (25th, 75th percentile)

bw, bodyweight; 1RM, one repetition maximum

Supplemental Table 7. Exercise intervention data summary by week.

Endurance Exercise Training									
**Week**	**2**	**3**	**4**	**5**	**6**	**7**	**8**	**9**	**10**
N	50	44	37	34	32	28	24	19	18
Per Protocol	42	35	35	31	27	25	20	17	16
Attended (d/wk)	3 (3, 3)	3 (3, 3)	3 (3, 3)	3 (3, 3)	3 (3, 3)	3 (3, 3)	3 (3, 3)	3 (3, 3)	3 (3, 3)
% HRR	66 (63, 69)	66 (64, 69)	67 (66, 70)	74 (71, 76)	74 (72, 76)	74 (71, 77)	73 (69, 76)	75 (72, 78)	76 (72, 80)
Duration (min.)	50 (50, 51)	60 (60, 60)	60 (60, 60)	60 (60, 61)	60 (60, 60)	60 (60, 60)	60 (60, 60)	60 (60, 60)	60 (60, 60)
MET min/week	677 (584, 807)	825 (734, 981)	843 (713, 1072)	933 (799, 1150)	911 (682, 1022)	912 (801, 1096)	900 (792, 1153)	969 (827, 1173)	1072 (861, 1173)
**Resistance Exercise Training**									
**Week**	**2**	**3**	**4**	**5**	**6**	**7**	**8**	**9**	**10**
N	54	51	45	41	38	35	30	25	23
Per Protocol	44	37	43	36	33	31	26	22	23
Attended (d/wk)	3 (3, 3)	3 (3, 3)	3 (3, 3)	3 (3, 3)	3 (3, 3)	3 (3, 3)	3 (3, 3)	3 (3, 3)	3 (3, 3)
Repetitions	766 (708, 801)	759 (711, 802)	760 (725, 794)	767 (699, 793)	752 (676, 786)	726 (688, 788)	734 (608, 788)	750 (725, 787)	752 (716, 787)
Sets	72 (72, 72)	72 (72, 72)	72 (72, 72)	72 (72, 72)	72 (68, 72)	72 (67, 72)	72 (61, 72)	72 (72, 72)	72 (72, 72)
Total load (kg/wk)	25055 (17721, 33247)	23726 (18432, 36599)	27963 (18869, 36882)	29584 (18097, 41744)	28022 (19125, 40508)	28106 (19823, 35020)	29374 (18496, 34600)	29939 (20866, 36519)	32002 (22279, 45378)
Statistics shown are median (25th, 75th percentile)									

Endurance Exercise Training									
**Week**	**2**	**3**	**4**	**5**	**6**	**7**	**8**	**9**	**10**
Week 1 is excluded as it serves as the introductory week. Weeks 11 and 12 are excluded because participants are completing phenotypic assessments or familiarization sessions in lieu of some intervention sessions									
HRR, Heart Rate Reserve; METmin, Metabolic equivalent of task-minutes									

Supplemental Table 8. Baseline and follow-up characteristics of participants with follow-up phenotypic data by randomized intervention group.

	EE (n=19)				RE (n=22)				Control (n=13)			
	**Baseline**	**Follow-up**	**Absolute Change**	**Percent Change**	**Baseline**	**Follow-up**	**Absolute Change**	**Percent Change**	**Baseline**	**Follow-up**	**Absolute Change**	**Percent Change**
Anthropometrics												
Weight (kg)	71.2 (63.8, 88.2)	71.2 (64.6, 85.1)	0.4 (-1.3, 2.2)	0.7 (-1.9, 3.1)	77.2 (66.6, 86.9)	80.4 (66.9, 87.1)	1.6 (-0.4, 2.2)	1.9 (-0.6, 3.0)	71.2 (67.2, 79.7)	74.3 (68.4, 81.2)	1.5 (1.2, 2.8)	2.3 (1.5, 4.4)
BMI ($kg \cdot m^{-2}$)	26.7 (23.6, 29.0)	26.9 (23.9, 28.6)	0.2 (-0.4, 0.8)	0.7 (-1.9, 3.1)	27.7 (24.7, 30.0)	28.0 (24.8, 30.1)	0.5 (-0.2, 0.7)	1.9 (-0.6, 3.0)	26.7 (24.8, 28.3)	27.5 (25.4, 29.6)	0.6 (0.4, 1.2)	2.3 (1.5, 4.3)
Waist circumference (cm)	91 (84, 98)	86 (83, 94)	-2 (-6, 0)	-2 (-6, 0)	94 (87, 102)	95 (85, 103)	-2 (-4, 3)	-2 (-4, 3)	90 (84, 96)	92 (92, 99)	4 (0, 5)	4 (0, 7)
Resting heart rate (bpm)	61 (54, 66)	61 (51, 68)	-1 (-4, 1)	-2 (-6, 2)	60 (55, 63)	60 (55, 69)	0 (-3, 7)	1 (-5, 11)	58 (51, 66)	56 (53, 67)	1 (0, 5)	2 (0, 9)
Resting systolic BP (mmHg)	116 (114, 123)	118 (110, 129)	2 (-4, 5)	1 (-4, 4)	112 (107, 119)	115 (109, 122)	1 (-3, 5)	1 (-3, 4)	113 (107, 126)	118 (111, 128)	3 (-2, 7)	3 (-2, 7)
Resting diastolic BP (mmHg)	72 (67, 75)	72 (68, 76)	-2 (-3, 1)	-2 (-5, 1)	71 (63, 78)	72 (62, 77)	0 (-3, 2)	-1 (-4, 3)	72 (68, 81)	74 (70, 78)	0 (-6, 4)	0 (-8, 5)
Biomarkers												
HbA1c (%)	5.3 (5.1, 5.6)	5.2 (5.1, 5.6)	0.0 (-0.1, 0.1)	0.8 (-1.3, 1.8)	5.3 (5.2, 5.6)	5.4 (5.2, 5.7)	0.0 (-0.1, 0.2)	0.0 (-1.7, 3.3)	5.3 (5.2, 5.5)	5.4 (5.2, 5.5)	0.0 (0.0, 0.1)	0.0 (-0.5, 1.9)
Glucose ($mg \cdot dL^{-1}$)	89 (84, 96)	88 (81, 94)	0 (-4, 4)	-1 (-5, 6)	90 (84, 94)	93 (89, 98)	4 (-4, 10)	4 (-4, 10)	91 (87, 94)	89 (83, 93)	-3 (-7, 6)	-3 (-7, 7)
Triglycerides ($mg \cdot dL^{-1}$)	92 (72, 118)	60 (53, 91)	-16 (-35, -10)	-20 (-43, -13)	88 (76, 122)	84 (68, 116)	-9 (-21, 15)	-8 (-22, 23)	76 (54, 97)	61 (54, 84)	-4 (-17, 4)	-9 (-21, 6)
Total cholesterol ($mg \cdot dL^{-1}$)	191 (175, 230)	190 (143, 220)	-16 (-28, 0)	-7 (-17, 0)	187 (166, 217)	184 (167, 201)	-4 (-18, 8)	-2 (-8, 4)	202 (165, 234)	181 (164, 225)	-10 (-24, -1)	-4 (-9, -1)
HDL cholesterol ($mg \cdot dL^{-1}$)	68 (48, 72)	56 (47, 64)	-6 (-8, 1)	-9 (-13, 1)	54 (43, 66)	56 (44, 64)	-3 (-8, 2)	-5 (-12, 5)	66 (61, 75)	62 (57, 74)	-4 (-7, -1)	-7 (-10, -2)
LDL cholesterol ($mg \cdot dL^{-1}$)	118 (92, 139)	110 (84, 142)	-8 (-13, 8)	-7 (-13, 8)	109 (98, 127)	114 (98, 126)	2 (-11, 10)	2 (-9, 11)	124 (90, 158)	106 (94, 147)	-8 (-12, 6)	-6 (-10, 7)

	EE (n=19)				RE (n=22)				Control (n=13)			
	**Baseline**	**Follow-up**	**Absolute Change**	**Percent Change**	**Baseline**	**Follow-up**	**Absolute Change**	**Percent Change**	**Baseline**	**Follow-up**	**Absolute Change**	**Percent Change**
VLDL cholesterol ($mg \cdot dL^{-1}$)	16 (13, 25)	12 (8, 14)	-6 (-8, -3)	-28 (-61, -18)	16 (14, 20)	15 (14, 20)	-2 (-4, 2)	-7 (-18, 20)	15 (11, 24)	12 (10, 18)	-2 (-4, 0)	-16 (-27, -1)
Hematocrit (%)	41.8 (39.6, 43.2)	37.9 (37.2, 40.0)	-2.2 (-3.4, -1.4)	-5.3 (-8.3, -3.8)	41.8 (39.9, 42.7)	41.2 (39.1, 42.8)	0.1 (-1.6, 1.2)	0.2 (-3.8, 2.6)	41.0 (39.5, 42.7)	40.4 (39.4, 41.8)	-0.7 (-1.7, 0.9)	-1.6 (-4.2, 2.2)
Thyroid stimulating hormone ($mIU \cdot L^{-1}$)	1.8 (1.6, 2.2)	2.0 (1.6, 3.0)	0.3 (0.0, 0.8)	13.1 (0.8, 37.5)	1.4 (1.1, 2.0)	1.8 (0.9, 2.4)	0.1 (-0.2, 0.7)	12.9 (-12.5, 55.4)	1.6 (1.0, 2.4)	1.6 (1.1, 2.3)	0.0 (-0.1, 0.5)	-1.4 (-6.4, 42.0)
Creatinine ($mg \cdot dL^{-1}$)	0.80 (0.62, 0.90)	0.78 (0.68, 0.90)	0.01 (-0.02, 0.08)	1.35 (-2.00, 10.96)	0.80 (0.70, 0.90)	0.82 (0.70, 0.90)	0.00 (-0.04, 0.02)	0.00 (-4.59, 2.90)	0.80 (0.64, 0.90)	0.85 (0.73, 0.92)	0.03 (0.00, 0.10)	4.52 (0.00, 12.50)
eGFR ($mL \cdot min^{-1} \cdot 1.73m^{-2}$)	107 (93, 121)	102 (86, 121)	-1 (-4, 2)	-1 (-4, 2)	102 (93, 110)	104 (93, 111)	0 (-3, 3)	0 (-2, 3)	103 (89, 105)	96 (82, 103)	-1 (-4, 0)	-1 (-4, 0)
Cardiopulmonary Exercise Testing												
VO_2peak ($L \cdot min^{-1}$)	1.63 (1.46, 1.92)	1.98 (1.66, 2.25)	0.25 (0.14, 0.39)	14.73 (9.48, 18.00)	1.63 (1.26, 2.35)	1.88 (1.48, 2.41)	0.11 (0.03, 0.24)	8.47 (0.93, 14.21)	1.53 (1.24, 1.67)	1.47 (1.34, 1.68)	0.00 (-0.06, 0.08)	0.26 (-4.04, 4.12)
VO_2peak ($mL \cdot kg^{-1} \cdot min^{-1}$)	22.2 (19.8, 27.1)	26.0 (22.8, 32.8)	3.9 (1.9, 4.7)	15.1 (10.7, 20.1)	23.8 (17.5, 30.9)	24.4 (18.2, 30.8)	1.1 (0.1, 3.2)	5.9 (0.4, 16.2)	21.9 (18.5, 26.5)	20.7 (18.0, 27.1)	-0.3 (-1.7, 0.6)	-1.6 (-6.2, 3.1)
Peak ventilation ($L \cdot min^{-1}$)	50.3 (47.8, 57.7)	61.4 (54.0, 70.7)	8.7 (3.9, 15.1)	14.0 (8.8, 27.1)	54.3 (38.2, 79.0)	58.6 (45.8, 77.3)	-0.5 (-3.9, 7.3)	-1.1 (-7.2, 12.1)	50.0 (42.7, 62.0)	49.4 (42.0, 71.2)	-1.9 (-7.6, -0.2)	-3.7 (-14.3, -0.6)
Peak O_2 pulse ($mL \cdot beat^{-1}$)	9.8 (9.0, 11.4)	11.5 (10.6, 13.6)	1.6 (1.1, 2.3)	17.1 (10.7, 20.3)	9.3 (7.7, 12.7)	10.5 (9.1, 13.1)	1.1 (0.5, 1.4)	11.2 (4.4, 17.0)	8.8 (8.4, 11.2)	8.9 (7.8, 10.3)	0.1 (-0.6, 0.8)	0.8 (-7.0, 8.6)
Peak RER	1.18 (1.16, 1.21)	1.16 (1.10, 1.22)	-0.01 (-0.06, 0.01)	-0.86 (-5.13, 0.85)	1.13 (1.07, 1.22)	1.15 (1.06, 1.20)	0.00 (-0.06, 0.02)	0.00 (-5.46, 1.91)	1.19 (1.13, 1.26)	1.16 (1.10, 1.23)	-0.01 (-0.03, 0.01)	-0.99 (-2.38, 0.87)
Peak workload (W)	140 (115, 170)	164 (134, 198)	28 (15, 34)	20 (12, 25)	130 (105, 182)	155 (117, 187)	12 (9, 24)	9 (5, 18)	131 (105, 145)	125 (105, 135)	-5 (-15, 2)	-4 (-9, 1)
Peak heart rate (bpm)	173 (157, 194)	170 (161, 186)	-4 (-9, 2)	-2 (-4, 1)	177 (167, 186)	174 (162, 184)	0 (-7, 0)	0 (-4, 0)	169 (160, 179)	164 (147, 175)	-3 (-7, 0)	-2 (-4, 0)
Systolic BP at termination (mmHg)	170 (159, 195)	171 (152, 186)	-6 (-14, 8)	-3 (-8, 4)	159 (150, 180)	168 (146, 192)	-3 (-8, 7)	-2 (-5, 4)	182 (145, 188)	170 (160, 192)	-8 (-20, 10)	-4 (-11, 6)

	EE (n=19)				RE (n=22)				Control (n=13)			
	**Baseline**	**Follow-up**	**Absolute Change**	**Percent Change**	**Baseline**	**Follow-up**	**Absolute Change**	**Percent Change**	**Baseline**	**Follow-up**	**Absolute Change**	**Percent Change**
Diastolic BP at termination (mmHg)	80 (70, 87)	79 (70, 84)	-3 (-8, 2)	-3 (-9, 2)	78 (66, 88)	79 (74, 84)	0 (-4, 2)	0 (-5, 3)	84 (80, 88)	86 (76, 90)	0 (-4, 2)	0 (-6, 2)
Overall RPE at termination	18 (17, 19)	19 (17, 20)	0 (-1, 1)	0 (-4, 6)	17 (17, 19)	19 (17, 20)	1 (0, 2)	6 (0, 12)	19 (17, 19)	19 (19, 19)	0 (0, 1)	0 (0, 6)
Physical measures												
Leg strength (Nm)[1]	139 (128, 186)	138 (116, 159)	-1 (-16, 11)	-1 (-10, 7)	140 (114, 193)	158 (118, 209)	15 (-4, 29)	12 (-3, 21)	138 (128, 163)	128 (124, 183)	-4 (-11, 5)	-3 (-8, 3)
Hand grip strength (kg)[2]	28 (23, 38)	28 (24, 32)	2 (-2, 3)	8 (-7, 12)	26 (22, 38)	30 (25, 42)	0 (-1, 3)	0 (-4, 11)	26 (22, 34)	26 (20, 38)	2 (-2, 3)	7 (-9, 10)
Statistics shown are median (25th, 75th percentile)												
[1]*The maximum of three peak torques obtained through three maximal voluntary contraction trials lasting 5 seconds each*												
[2]*The maximum of three grip strength trials which occur after one submaximal practice trial to familiarize the participant with the feel of the instrument*												
BMI, body mass index; BP, blood pressure; HDL, high-density lipoprotein; HbA1c, hemoglobin A1c; LDL, low-density lipoprotein; RPE, rating of perceived exertion; VLDL, very low-density lipoprotein; VO_2, volume of oxygen; W, watts; eGFR, estimated glomerular filtration rate												

Supplemental Table 9. Baseline and follow-up endurance exercise acute bout parameters.

Variable	Baseline N = 14	Follow-up N = 14	Absolute Change N = 14	Percent Change N = 14
Median (25th, 75th percentile)				
Workload (W)	57 (46, 70)	82 (64, 112)	20 (14, 42)	30 (24, 82)
Total Work (kJ)	131 (103, 168)	162 (125, 238)	36 (26, 89)	25 (22, 52)
RPM	71 (68, 76)	75 (71, 77)	1 (-2, 4)	2 (-3, 5)
VO_2 (% peak)	65.5 (64.6, 66.7)	64.8 (61.6, 66.6)	-2.0 (-6.0, 0.7)	-3.0 (-9.2, 1.0)
VO_2 ($L \cdot min^{-1}$)	1.12 (0.94, 1.27)	1.24 (1.04, 1.36)	0.12 (0.07, 0.20)	9.54 (6.91, 14.51)
VO_2 ($mL \cdot kg^{-1} \cdot min^{-1}$)	14.5 (12.8, 18.2)	16.0 (15.3, 20.4)	1.7 (1.3, 2.4)	9.5 (7.9, 13.1)
Carbohydrate ($g \cdot min^{-1}$)	0.88 (0.78, 1.17)	1.18 (0.87, 1.62)	0.14 (0.05, 0.35)	13.62 (7.57, 31.78)
Fat ($g \cdot min^{-1}$)	0.1 (0.1, 0.3)	0.2 (0.1, 0.2)	0.0 (-0.1, 0.1)	9.8 (-42.8, 49.7)
Carbohydrate ($kcals \cdot min^{-1}$)	3.6 (3.2, 4.8)	4.8 (3.6, 6.6)	0.6 (0.2, 1.4)	13.6 (7.6, 31.8)
Fat ($kcals \cdot min^{-1}$)	1.4 (1.2, 2.5)	1.7 (0.5, 2.3)	0.2 (-0.6, 0.8)	9.8 (-42.8, 49.7)
Carbohydrate (%)	73 (58, 80)	76 (54, 91)	0 (-7, 11)	-1 (-11, 19)
VE ($L \cdot min^{-1}$)	27.5 (24.1, 30.7)	33.2 (27.6, 42.9)	5.7 (2.7, 15.5)	20.9 (9.2, 43.0)
RER	0.92 (0.87, 0.94)	0.92 (0.86, 0.98)	0.00 (-0.02, 0.04)	-0.11 (-2.26, 4.16)
Heart rate (bpm)	135 (129, 144)	141 (133, 155)	0 (-6, 12)	0 (-4, 9)
Heart rate reserve (%)	67 (60, 71)	74 (64, 74)	7 (-1, 14)	10 (-2, 21)
O_2 Pulse ($mL \cdot beat^{-1}$)	8.1 (7.0, 9.6)	8.7 (8.0, 10.6)	0.8 (0.2, 1.7)	9.2 (1.3, 22.2)

RER, respiratory exchange ratio; RPM, revolutions per minute; VE, ventilation; VO_2, volume of oxygen; W, watts

Supplemental Table 10. Baseline and follow-up resistance exercise acute bout parameters.

Characteristic	Baseline N = 18	Follow-up N = 18	Absolute Change N = 18	Percent Change N = 18
Median (25th, 75th percentile)				
Acute bout duration (minutes)	54.6 (51.4, 60.8)	52.7 (49.6, 56.2)	-1.5 (-6.8, 0.8)	-2.9 (-11.5, 1.4)
1RM				
Leg press (kg)	123 (86, 184)	154 (116, 225)	29 (14, 45)	26 (6, 59)
Chest press (kg)	32 (25, 45)	44 (39, 66)	13 (6, 25)	29 (17, 52)
Leg extension (kg)	67 (43, 88)	70 (54, 106)	16 (10, 28)	29 (12, 44)
1RM, normalized				
Leg press ($kg\cdot bw^{-1}$)	1.61 (1.31, 2.11)	1.99 (1.66, 3.15)	0.48 (0.13, 0.62)	27.08 (3.64, 48.59)
Chest press ($kg\cdot bw^{-1}$)	0.41 (0.36, 0.64)	0.64 (0.48, 0.96)	0.15 (0.09, 0.32)	28.42 (15.15, 48.01)
Leg extension ($kg\cdot bw^{-1}$)	0.99 (0.64, 1.12)	0.99 (0.77, 1.27)	0.21 (0.11, 0.33)	28.37 (9.35, 41.31)
Percent 1RM				
Leg press ($kg\cdot 1RM^{-1}$)	63 (56, 69)	68 (63, 73)	8 (-9, 12)	12 (-12, 20)
Chest press ($kg\cdot 1RM^{-1}$)	58 (52, 67)	63 (60, 66)	6 (-7, 13)	11 (-10, 24)
Leg extension ($kg\cdot 1RM^{-1}$)	57 (49, 67)	57 (50, 63)	-2 (-10, 6)	-3 (-16, 14)
Average resistance				
Chest press (kg)	19 (14, 32)	27 (23, 41)	8 (3, 13)	51 (17, 74)
Overhead press (kg)	8 (5, 19)	15 (8, 28)	4 (2, 9)	55 (33, 92)
Seated row (kg)	22 (18, 34)	32 (25, 44)	7 (6, 10)	30 (21, 44)
Triceps extension (kg)	21 (12, 27)	28 (17, 42)	7 (3, 10)	33 (20, 48)
Biceps curl (kg)	12 (5, 22)	18 (11, 32)	6 (3, 9)	41 (24, 64)

Characteristic	Baseline N = 18	Follow-up N = 18	Absolute Change N = 18	Percent Change N = 18
Leg press (kg)	78 (56, 114)	104 (71, 145)	18 (12, 44)	32 (19, 40)
Leg curl (kg)	45 (32, 54)	48 (41, 58)	7 (5, 13)	18 (13, 29)
Leg extension (kg)	41 (23, 54)	48 (31, 57)	7 (5, 13)	29 (10, 35)
Average repetitions per set				
Chest press	8.83 (8.00, 9.33)	10.00 (9.33, 11.00)	1.50 (0.67, 2.67)	16.93 (7.28, 30.88)
Overhead press	9.00 (7.33, 10.00)	9.83 (9.00, 10.33)	0.67 (-1.08, 1.58)	8.76 (-10.81, 19.33)
Seated row	10.17 (9.33, 11.00)	10.00 (9.67, 11.33)	-0.33 (-1.33, 1.33)	-3.23 (-11.87, 14.05)
Triceps extension	10.33 (9.67, 11.00)	10.17 (9.33, 10.67)	-0.17 (-1.00, 0.58)	-1.52 (-9.24, 5.93)
Biceps curl	9.67 (8.00, 10.00)	9.67 (9.33, 10.33)	0.00 (-0.25, 1.00)	0.00 (-2.50, 11.24)
Leg press	11.17 (9.67, 12.67)	10.83 (10.33, 11.67)	0.00 (-1.50, 1.75)	0.70 (-12.86, 15.91)
Leg curl	9.67 (9.00, 11.00)	10.17 (9.67, 11.00)	0.00 (-0.67, 0.67)	0.00 (-7.07, 7.34)
Leg extension	9.50 (8.67, 11.00)	10.67 (10.00, 11.67)	1.00 (0.33, 1.83)	9.23 (3.59, 14.81)
Overall	9.7 (9.4, 10.1)	10.3 (9.8, 10.5)	0.7 (-0.1, 0.9)	6.7 (-1.3, 9.3)
Total load				
Upper body (kg)	2,637 (1,588, 3,741)	3,778 (3,005, 4,997)	1,195 (758, 1,755)	44 (30, 86)
Lower body (kg)	5,069 (3,626, 7,466)	6,612 (4,783, 7,600)	1,566 (793, 2,152)	28 (18, 39)
Combined (kg)	7,620 (5,610, 11,530)	10,624 (7,627, 11,891)	3,108 (1,657, 3,978)	32 (27, 45)
Total load, normalized				
Upper body ($kg \cdot bw^{-1}$)	39 (25, 45)	49 (43, 68)	16 (9, 24)	45 (26, 83)

Characteristic	Baseline<br>N = 18	Follow-up<br>N = 18	Absolute Change<br>N = 18	Percent Change<br>N = 18
Lower body ($kg \cdot bw^{-1}$)	62<br>(58, 85)	84<br>(69, 107)	19<br>(10, 25)	27<br>(16, 40)
Combined ($kg \cdot bw^{-1}$)	101<br>(84, 131)	132<br>(114, 180)	36<br>(22, 50)	30<br>(25, 48)

bw, bodyweight; 1RM, one repetition maximum

Supplemental Table 11. Overview of biospecimen collection success for each sample type at baseline and follow-up.

	Baseline Acute Test				Follow-Up Acute Test			
**Sample Type**	**# Expected**	**# Collected (% Collected)**	**# Collected on Time (% Collected)**	**# Adequate Yield for CAS Platforms (% Collected)**	**# Expected**	**# Collected (% Collected)**	**# Collected on Time (% Collected)**	**# Adequate Yield for CAS Platforms (% Collected)**
Muscle	438	413 (94)	392 (95)	338 (82)	94	78 (83)	76 (97)	67 (86)
Adipose	352	342 (97)	332 (97)	334 (98)	78	69 (88)	67 (97)	67 (97)
PAXGene RNA	910	876 (96)	813 (93)	876 (100)	171	156 (91)	147 (94)	156 (100)
EDTA SS Plasma	910	876 (96)	813 (93)	876 (100)	171	156 (91)	147 (94)	156 (100)
EDTA Packed Cells	910	876 (96)	813 (93)	875 (100)	171	156 (91)	147 (94)	156 (100)
PBMC	910	865 (95)	807 (93)	862 (100)	171	155 (91)	146 (94)	155 (100)
EDTA Packed Cells DMSO	910	876 (96)	813 (93)	---	171	156 (91)	147 (94)	---
EDTA DS Plasma	435	424 (97)	400 (94)	---	93	87 (94)	85 (98)	---
Heparin Plasma	910	867 (95)	809 (93)	---	171	155 (91)	146 (94)	---
Serum	176	173 (98)	164 (95)	---	45	44 (98)	44 (100)	---

	Baseline Acute Test				Follow-Up Acute Test			
**Sample Type**	**# Expected**	**# Collected (% Collected)**	**# Collected on Time (% Collected)**	**# Adequate Yield for CAS Platforms (% Collected)**	**# Expected**	**# Collected (% Collected)**	**# Collected on Time (% Collected)**	**# Adequate Yield for CAS Platforms (% Collected)**
RNA, Ribonucleic Acid; EDTA, Ethylenediaminetetraacetic Acid; SS Plasma, Single Spin Plasma; PBMC, Peripheral Blood Mononuclear Cells; DMSO, Dimethyl Sulfoxide; DS Plasma, Double Spin Plasma; CAS, Chemical Analysis Sites								
PAXGene RNA is generated from the PAXGene RNA tube; SS Plasma, Packed Cells, and Packed Cells DMSO are generated from the SS EDTA tube; DS Plasma is generated from the DS EDTA Tube; Heparin Plasma and PBMCs are generated from the Cell Preparation Tube; Serum is generated from the Serum tube								
*# Expected Collections = Number of collections expected for acute tests performed<br># Collected = Number of collections that resulted in at least one processed sample vial<br># Collected on Time = Number of collections that resulted in at least one processed sample vial and was collected within the allowable time range<br>% Collected = (# Collected/# Expected Collections)*100<br>% Collected on Time = (# Collected on Time/# Collected)*100<br>% Muscle and Adipose Collected with Adequate Yield = (# Collected with Adequate Yield for Shipment to CAS Platforms/# Collected)*100<br>% Blood Collected with Adequate Yield = (# Collected with Adequate Yield for Shipment to CAS Platforms/# Collected)*100; Sample types without data have not shipped to Chemical Analysis Site*								
